# Organizational supports used by private child and family serving agencies to facilitate evidence use: a mixed methods study protocol

**DOI:** 10.1186/s13012-017-0580-1

**Published:** 2017-04-08

**Authors:** Emmeline Chuang, Crystal Collins-Camargo, Bowen McBeath

**Affiliations:** 1 Department of Health Policy and Management, Fielding School of Public Health, University of California, Los Angeles, 650 Charles E Young Dr. South, Los Angeles, CA 90095-1772 USA; 2 Kent School of Social Work, University of Louisville, 2217 S. Third Street, Louisville, KY 40292 USA; 3 School of Social Work, Portland State University, PO Box 751, Portland, OR 97207 USA

**Keywords:** Children and adolescents, Mixed methods, Evidence use, Organizational supports

## Abstract

**Background:**

Challenges to evidence use are well documented. Less well understood are the formal supports—e.g., technical infrastructure, inter-organizational relationships—organizations may put in place to help overcome these challenges. This study will identify supports for evidence use currently used by private child and family serving agencies delivering publicly funded behavioral health and/or human services; examine contextual, organizational, and managerial factors associated with use of such supports; and determine how identified supports affect evidence use by staff at multiple levels of the organization.

**Methods:**

We will use a sequential explanatory mixed methods design, with study activities occurring in two sequential phases: In phase 1, quantitative survey data collected from managers of private child and family serving agencies in six states (CA, IN, KY, MO, PA, and WI) and analyzed using both regression and qualitative comparative analysis (QCA) will identify organizational supports currently being used to facilitate evidence use and examine the contextual, organizational, and managerial factors associated with the use of such supports. In phase 2, data from phase 1 will be used to select a purposive sample of 12 agencies for in-depth case studies. In those 12 agencies, semi-structured interviews with key informants and managers, focus groups with frontline staff, and document analysis will provide further insight into agencies’ motivation for investing in organizational supports for evidence use and the facilitators and barriers encountered in doing so. Semi-structured interviews with managers and focus groups with frontline staff will also assess whether and how identified supports affect evidence use at different levels of the organization (senior executives, middle managers, frontline supervisors, and frontline staff). Within- and between-case analyses supplemented by QCA will identify combinations of factors associated with the highest and lowest levels of staff evidence use.

**Discussion:**

This study will inform efforts to improve sustainment, scale-up, and spread of evidence by providing insight into organizational and managerial strategies that facilitate evidence use, the contexts in which these strategies are most effective, and their effect on evidence use by staff at different levels of the organization.

## Background

Effective use of research evidence, or knowledge that has been subjected to testing and found to be credible [1, 2], by health and human service practitioners can reduce disparities in the costs and quality of care, enhance service effectiveness and worker satisfaction, and improve outcomes experienced by vulnerable children and families [3–6]. Recognizing the importance of research evidence for improving organizational performance and service outcomes, policymakers in many countries have made evidence-informed health and human services a priority [7, 8]. In the USA, an increasing number of state and local governments now link the use of research evidence regarding “effective” programs and practices, including evidence-based treatments (EBTs), to funding decisions or service reimbursement [9].

Despite the importance of research evidence use for achieving positive outcomes for vulnerable children and families, research-to-practice gaps persist across settings, conditions, and population groups [10]. In health care, for example, only about half of recommended care practices are implemented [11, 12]. Uptake of research recommendations is even lower for prevention and behavior change programs and for managerial practices such as goal setting and performance feedback [13, 14]. In the human services sector, national survey evidence suggests that only 25% of child welfare agency programs and practices are evidence based [15]. In all states, an increasingly large proportion of publicly funded services for child welfare-involved children and families are delivered via purchase of service contracts with private agencies [16, 17]. Although these private agencies are heterogeneous as a population [18], the majority provide both behavioral health and human services to vulnerable children and families [19]. However, only a small proportion of agency administrators and practitioners report regularly using research to inform their daily work [20, 21].

Challenges to the use of research evidence, and particularly to the adoption, implementation, and sustainment of EBTs, are well documented [8, 22, 23]. Less studied are the formal supports—i.e., staff positions, infrastructural supports, and special initiatives—organizations may put in place to help overcome these challenges. For example, in some clinical settings, knowledge broker positions (i.e., intermediaries accountable for encouraging knowledge use) have successfully been used to support evidence-informed decision-making [24]. Ties to opinion leaders and researchers outside of the organization [25, 26], a supportive technical infrastructure [27, 28] and an organizational climate that rewards evidence use [24, 29] have also been identified as affecting evidence use.

When present, these supports may signify an organizational commitment to improving service quality and program outcomes. However, these supports typically require significant managerial and workforce investment and can be costly to develop, implement, and sustain. Agencies may vary in their capacity and/or willingness to utilize such supports and in their approach to systematizing the use of research evidence by managers and other staff. Currently, we know little about the extent to which health and human service agencies use such supports to facilitate evidence use by staff. We also know little about the contextual and organizational factors associated with agency use of such supports or the extent to which these supports affect evidence use by staff at different levels of the organization.

The current study will address this gap by collecting qualitative and quantitative data from private child and family serving agency managers and staff to address the following aims:

- Aim 1: Identify formal organizational supports used by private child and family serving agencies to facilitate evidence use
- Aim 2: Examine contextual, organizational, and managerial factors affecting agency use of formal supports
- Aim 3: Determine how formal organizational supports affect use of research evidence at multiple levels of the organization

### Conceptual framework

**Fig. 1:**
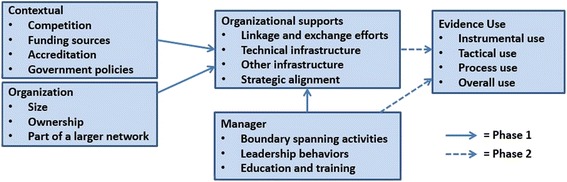
Conceptual model of organizational supports used by private agencies to facilitate evidence use

Study activities will be guided by the conceptual framework in Fig. 1. This model was developed following a review of the literature on evidence use in the human services, public health, and associated scholarly domains and is informed by concepts from resource dependence theory [30], institutional theory [31, 32], theories of leadership [33, 34], and the strategic management literature [35, 36].

### Organizational supports that facilitate evidence use

We propose to examine four types of formal supports that agencies may use to facilitate evidence use. These supports include *linkage and exchange efforts*, *technical infrastructure*, *other knowledge management infrastructure*, and *strategic alignment*.

Linkage and exchange efforts refer to formal ties to knowledge brokers [37] outside of the agency who can assist in acquiring, assessing, adapting, or applying evidence in decision-making or practice [38–41]. These knowledge brokers can include researchers, professional associations, consultants, or research use networks. The use of knowledge brokers to assist with distilling and disseminating research to practitioners is increasingly common in Canada and the UK [42–44] and has also been seen increased uptake in the US health care sector [45] but has not been systematically examined in the US behavioral health or human service sectors.

Technical infrastructure includes internal data systems and/or other tools designed to facilitate access to and use of evidence by agency staff [46–48]. Research suggests that investment in technical infrastructure may be necessary to facilitate evidence use [49, 50]. At a basic level, agencies may provide staff with computers that permit use of free online resources or develop virtual libraries to promote staff access to research evidence. Agencies may also invest in performance measurement systems designed to collect and report data on program- or agency-level performance indicators or in client management information systems that provide real-time data on client service utilization and outcomes.

Other knowledge management infrastructure includes other agency resources allocated for the purpose of building capacity to use or promote evidence use. These supports include having formal positions (full or part-time) responsible for supporting evidence use, e.g., internal knowledge brokers or other staff formally assigned to retrieve, translate, and disseminate research knowledge within the agency or research agency programs and initiatives. Agencies may also promote staff training and continuing education on specific research topics.

Finally, strategic alignment [51] refers to other formal efforts intended to establish an organizational culture and climate, i.e., workers’ perceptions of norms and expectations in their work environment [52, 53], that prioritizes evidence use. Examples include emphasis on the importance of evidence use in the agency’s mission or strategic plan or establishing policies and practices that promote accountability for evidence use (e.g., incorporating a requirement for research evidence use into staff performance reviews or compensation plans).

### Factors hypothesized to affect agency use of formal organizational supports

As shown in Fig. 1, we conceptualize agency use of formal organizational supports to facilitate evidence use as affected by factors at the environmental, agency, and managerial levels.

*Contextual and organizational factors* hypothesized to affect agency use of such supports are informed by two complementary macro-theoretical perspectives on organizational behavior, resource dependence theory and institutional theory [30, 31, 54]. Consistent with resource dependence theory and institutional theory, we posit that agencies will invest in formal organizational supports for evidence use if leaders perceive this investment as enhancing agencies’ ability to secure resources vital to organizational maintenance and survival (e.g., providing a competitive edge with clients or funders) [55, 56] or if they face institutional pressure to do so from funders, policy-makers, competitors, and accrediting and educational bodies whose support agencies need in order to be perceived as “legitimate” [57–59]. Specific factors hypothesized to affect agency investment in organizational supports for evidence use include inter-organizational competition [60], accreditation requirements promoting evidence-informed practice, state or local government policies requiring contractor use of evidence-based practice, and agency size [61, 62].

*Managerial characteristics* hypothesized to affect agency use of such supports are identified from the strategic management literature [35, 36], theories of leadership [33, 34], and prior research on evidence use in health care [38, 63, 64]. Specifically, we propose to examine three types of managerial characteristics hypothesized to affect agency use of formal organizational supports to facilitate evidence use: boundary spanning activities [65, 66] (i.e., management of relations with external entities), leadership behaviors [67] (e.g., the actions leaders take to motivate staff and implement plans), and managers’ education, training, and attitudes towards evidence use [38, 63, 64]. These characteristics, particularly leadership behaviors and attitudes towards evidence use, may also directly affect evidence use by agency staff.

## Methods/design

**Fig. 2:**
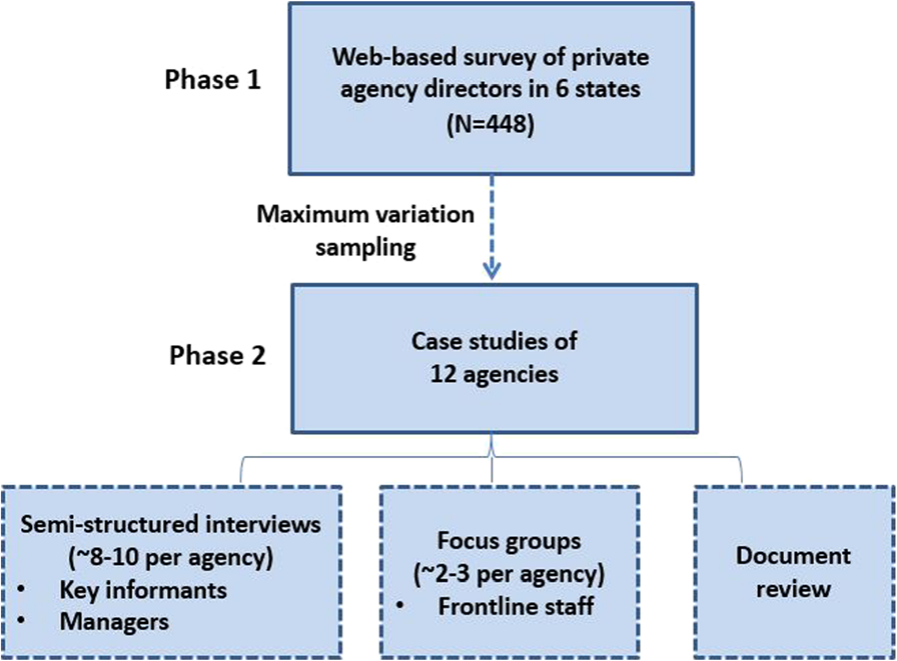
Overview of study design

We will use a sequential explanatory mixed methods design [68], with study activities occurring in two sequential phases: In phase 1, quantitative survey data collected from managers of private child and family serving agencies in six states will examine organizational supports currently being used by agencies to facilitate evidence use (aim 1) and identify contextual, organizational, and managerial factors associated with agency use of such supports (aim 2). In phase 2, data from phase 1 will be used to select a purposive sample of 12 agencies for in-depth case studies. In those 12 agencies, key informant interviews, focus groups, and document analysis will provide further insight into agencies’ motivation for investing in organizational supports for evidence use and the facilitators and barriers encountered in doing so (aim 2). Semi-structured interviews with managers (executives, middle managers, and frontline supervisors) and focus groups with frontline staff will examine whether and how identified supports affect evidence use at different levels of the organization (aim 3). An overview of our proposed study design is provided in Fig. 2.

### Phase 1: quantitative survey of private agency executives

In phase 1, we will administer a web-based survey to private child and family serving agency executives in six states that vary in size and sociopolitical context (CA, IN, KY, MO, PA, and WI). Included agencies in six states will enhance generalizability of study findings and allow us to better control for differences in local institutional context. It will also ensure that we have a sufficient sample size to conduct proposed analyses.

#### Sampling strategy and recruitment procedures

Eligible agencies in each state include all private agencies serving the child welfare population that are members of a state or national association of private providers. Prior evidence suggests that public agencies contract with such agencies for a broad spectrum of services, ranging from core child welfare services such as foster care and case management to behavioral health and health care services [18, 69]. In the absence of a comprehensive database identifying our study population, we will partner with the major membership associations representing private child and family serving agencies in state and federal policymaking to distribute survey invitations to their members, as well as private non-member agencies on their association listservs. Our partners include six state membership associations of private child and family serving agencies as well as the Alliance for Strong Families and Communities (the Alliance). Based on 2015 membership, we anticipate a sample size of approximately 448 agencies.

A single executive in each agency will receive an electronic letter of invitation that provides additional information on the study’s purpose and a hyperlink to the survey. Typically, this executive will be the individual identified by our study partners as their primary contact for that agency. Respondents will be asked to complete the survey only once per agency. To facilitate tracking, each agency will receive a unique survey link. The survey portal will remain open for 12 weeks. Upon completion of the survey, responding agencies will be entered in a raffle to win one of 45 $100 electronic gift cards (or equivalent donation to their agencies). Respondents will also be asked if their agency is interested in participating in the phase 2 of the study.

#### Survey instrument development

An overview of survey domains and content is provided in Table 1 reflecting measures previously validated either in child welfare or other health and human service contexts as well as prior research conducted by the study investigators [18, 70–75]. To ensure questions are of high salience to agency executives, the survey instrument will be piloted with an expert panel comprised of 5–6 private agency administrators and other key stakeholders from states not targeted for survey participation, and refined to ensure it takes no more than 20 min to complete.

**Table 1:** Overview of phase 1 director survey measures

Construct	Measure
Evidence use	
Use of research evidence	Four types of research use: *Instrumental use* refers to the direct use of research evidence in decision-making or in identifying a solution to a specific problem, e.g., in deciding to adopt or implement a specific program or practice or choosing a specific course of action with a client [63]. *Tactical use*, also known as *persuasive* or *symbolic use*, refers to the use of evidence to legitimize, justify, or otherwise mobilize support for actions or decisions [100]. *Process use* encompasses direct involvement in the design or conduct of research. Finally, *overall use* refers to the use of any kind of research in any way and encompasses all of the previous types of evidence use [101].
Formal organizational supports used to facilitate evidence use	
Linkage and exchange efforts	Ties to knowledge brokers outside the organization who can assist in acquiring, assessing, adapting, or applying evidence in decision-making or practice. Specific types of knowledge brokers to be examined include researchers, professional associations, advocacy groups, and consultants or other technical assistance providers.
Technical infrastructure	Data systems and/or other technical infrastructure designed to facilitate access to and use of evidence by agency staff, e.g., information systems that disseminate research evidence and/or provide timely data and feedback to staff on client utilization, experiences, or outcomes, etc.
Other knowledge management infrastructure	Other infrastructure designed to promote evidence use within the agency. Specifically, we will examine whether there are (1) formal positions accountable for supporting evidence use and/or (2) formal organizational policies or practices designed to develop agency capacity for evidence use; (3) staff directly involved in research and/or quality improvement activities
Strategic alignment	Emphasizing the importance of evidence use in the agency’s mission, vision, values and/or strategic plan and/or any other formal efforts to establish an organizational climate that prioritizes evidence use
Factors hypothesized to affect agency use of formal organizational supports	
Competition	Local competition for funding, staff, and clientele
Funding sources	Major sources of revenue, the percentage of revenues received directly from each source during the most recent fiscal year (e.g., Medicaid, state or county contracts with public child welfare agencies), and whether payment is linked to a performance-based accountability mechanism
Accreditation	Whether the agency is accredited by COA, CARF, JCAHO, or another accrediting body
Government policies	State and/or local requirements for the use of EBTs in services and/or for research evidence use
Agency size	Number of full-time staff or full-time staff equivalents
Organizational auspices	Whether the agency is not-for-profit or for-profit and whether the agency is part of a larger network or system
Boundary spanning activities	Number of hours per week spent engaging in each of the following activities with external stakeholders: (1) liaison activities with other monitoring or licensing organizations; (2) activities with professional associations; (3) consulting with and/or participating in task groups with other service providers; (4) activities with researchers or technical assistance providers (5) public presentations and appearance in the community; and (6) contributing to federal, state, and/or local policy making
Leadership behaviors	Respondents’ leadership style
Director education and training	Directors’ highest educational degree, length of time in current role, whether he/she ever completed a research class (e.g., on research design or statistics) and/or class on quality improvement techniques, attitudes towards evidence use, and personal use of research evidence

#### Analyses

The phase 1 unit of analysis is the private child and family serving agency. Factor analysis will examine the underlying factor structure of any quantitative measures not previously used in this context [76]. Univariate and bivariate analyses will descriptively examine the prevalence of different formal organizational supports to facilitate evidence use across the sample of private child welfare agencies (aim 1). Subsequent analyses will explore the extent to which contextual, agency, and managerial factors identified in Fig. 1 are associated with the presence and use of different organizational supports for evidence use (aim 2). These analyses will occur in three stages: First, multiple regression will identify contextual, organizational, and managerial factors significantly associated with agency use of organizational supports to facilitate evidence use.

Next, qualitative comparative analysis (QCA) will be used to identify specific combinations of contextual, agency, and managerial factors associated with high vs. low levels of organizational support for evidence use [77–79]. QCA is a set-theoretic method based on Boolean algebra that is increasingly used in sociological, management, and health services research to explore complex social phenomena [80, 81]. The primary benefit of QCA for the proposed research is the technique’s ability to identify conditions or combinations of conditions that are necessary vs. sufficient for agency investment in organizational supports for evidence use. A condition is considered *necessary* if it must be present in order for an outcome to occur; however, the presence of a necessary condition does not ensure the outcome will occur. For example, agencies may need to be a certain size in order to invest in management information systems or other technical infrastructure. However, even if agency size is necessary, it may not be sufficient for agency investment in technical infrastructure in the absence of other conditions such as institutional pressures or managerial support for evidence use. Conditions or combinations of conditions are considered *sufficient* if they consistently produce an outcome of interest when present. In our study, there may be multiple combinations of contextual, organizational, and managerial factors that are sufficient but not necessary for agency investment in organizational supports for evidence use. For example, having either an executive with a positive attitude towards research or funders that link evidence use to payments could each be sufficient to ensure agency investment in supports for evidence use. This assumption of causal heterogeneity, i.e., different combinations of conditions can lead to the same outcome [77], is a strength of QCA and will allow for development of models that better reflect the reality of complex organizational phenomena than if we relied solely on regression-based techniques.

Configurational solutions identified through QCA will be inputted into regression models to identify configurations of factors that are significantly associated with agency use of formal organizational supports even after controlling for other agency characteristics. Regression analyses will be conducted using Stata 13.0 [82]. QCA will be conducted using either the fsQCA software [78] or the FUZZY module in Stata [83], depending on the specific constructs being tested.

### Phase 2: mixed methods case studies of 12 agencies

In phase 2, our primary aim is to examine the ways in which identified organizational supports affect the use of evidence at multiple levels of the agency (aim 3). We will also explore in more detail the contextual, organizational, and managerial factors that may affect agency use of such supports and use of research at different levels of the agency (aim 2). To achieve this objective, we will employ a multiple case study design, with the agency as the unit of analysis.

#### Sampling strategy

In phase 2, eligible agencies include all private child and family serving agencies from phase 1 that expressed an interest in participating in phase 2. Eligible agencies will be stratified by prevalence of formal organizational supports for evidence use, and a maximum variation sampling procedure [84] will be used to identify a diverse sample of 12 private agencies for in-depth case study analysis. Within each of these 12 agencies, qualitative data will be collected from respondents at multiple levels of the organization (e.g., agency executives, middle managers, frontline supervisors, and frontline staff).

#### Semi-structured interviews with key informants and managers

We propose to conduct semi-structured qualitative interviews with key informants and managers at multiple levels (e.g., executive team, middle management, frontline supervisors) within each agency (~8–10 interviews per agency). Qualitative interviews with 2–3 key informants in each agency will provide insight into agency strategic priorities, motivation for investing in identified organizational supports, and facilitators and barriers to putting these supports in place. Qualitative interviews with managers (additional ~6–8 total) will provide insight into how respondents at different levels of the agency use research evidence and the ways in which available organizational supports affect evidence use. Interviews will also further explore contextual, organizational, and managerial factors—particularly leadership behaviors and staff training and resources—that may affect perceived utility of these organizational supports and overall evidence use (see Table 2).

**Table 2:** Overview of phase 2 qualitative domains

Domain	Type	Examples
Respondent role	Open-ended	• Both: Can you tell me a little bit about your role in [agency]? What does a typical day or week look like?
Organizational context	Open-ended	• KS: Can you tell me a little bit about the [agency]’s strategic priorities? • FLW: How much discretion do you have in your work with clients? What factors external or internal to your agency have the greatest influence on your daily work?
Evidence use	Closed-ended	• Measures of research evidence use will be drawn from one of three validated instruments: the Output scale of the Structured Interview of Evidence Use (SIEU) [102], the Evidence-Informed Practice Survey (EIPS) [103], or the Research Utilization Questionnaire (RUQ) [100, 104]. Specific measures administered will be based in part on expert panel feedback regarding topic salience and respondent burden.
	Open-ended	• KS: What types of information do you use when making decisions about programs or policies? Do you view certain types of evidence as more useful to this process than others? • FLW: What types of information or ‘evidence’ do you use when making decisions about clients? Do you view certain types of evidence as more useful to this process than others? Why or why not? What about in other aspects of your daily work?
Organizational supports for evidence use	Open-ended	• Both: To what extent does your agency value the use of evidence (particularly research evidence) in your day-to-day work? What gives you this impression? • Both: What types of supports does your agency have in place to facilitate evidence-informed decision making? How useful do you find these supports? Why or why not? How often do you take advantage of these supports? Are there other supports or resources you wish you had access to? Why or why not? • KS: How long have these supports been in place? What motivated your agency to invest in these supports? How effective have you found these supports at helping your agency fulfill its mission and/or accomplish its strategic priorities?
Leader facilitation of evidence use	Closed-ended	• Leadership style and effectiveness using the Multifactor Leadership Questionnaire Short Form (MLQ-5X) [33] • Boundary spanning activities adapted from the National Drug Abuse Treatment System Survey [105, 106]
	Open-ended	• KS: Can you provide an example of any actions you have taken to promote evidence use by staff? • Both: To what extent do leaders at your agency value the use of evidence in your daily work? What gives you this impression?
Respondent characteristics	Closed-ended	• Respondents’ training, knowledge, and skills in using research; specific measures will be drawn from previous studies of research use in the health care sector [64, 107] • Basic demographics (age, gender, race/ethnicity)

*KS* key stakeholder, *FLW* frontline worker

All interviews will last no longer than 45 min and will be conducted using a semi-structured interview guide tailored to the respondent’s role within the agency. With respondents’ permission, all interviews will be recorded and transcribed verbatim.

#### Focus groups with frontline staff

Focus groups rely on group interaction to generate insights and can be effective at encouraging participation from individuals reluctant to be interviewed on their own [85]. In phase 2, focus groups with frontline staff will examine the extent to which staff currently use research evidence and the ways in which identified organizational supports and leader behaviors do or do not affect evidence use (see Table 2). Depending on agency size, we anticipate conducting 2–3 focus groups per agency, with 5–8 participants per focus group. An experienced moderator will introduce the topics to be addressed, monitor dynamics of group discussion to ensure all views are represented, and ensure the discussion stays on track. Focus groups will be conducted in absence of other agency personnel, and the script will be tailored to create an atmosphere in which staff feel safe and comfortable sharing their opinions (see Table 2). A second researcher will be present to take detailed notes on the discussion. Focus groups will last approximately 90–100 min and, with respondents’ permission, will be recorded and transcribed verbatim.

#### Document analysis

As appropriate during the semi-structured interview process, key informants will be asked to provide access to documents that clarify organizational policies, priorities, or supports for evidence use. These documents will provide a low-cost method of augmenting respondents’ descriptions of their organizational contexts and available supports for evidence use [84, 86]. These documents may include but are not limited to program manuals, quality improvement plans and practice protocols, the agency’s response to a request for proposals that seeks funding for training or other infrastructure development activities, or publicly available information on the agency’s structure and services. With respondents’ and agencies’ permission, these documents will be uploaded into NVivo for analysis.

#### Analyses

Phase 2 analyses will occur in multiple stages. First, all qualitative data will be uploaded into the qualitative software QSR NVivo 10.0 for analysis [87]. Case study analyses of these qualitative data will be guided by pattern-matching logic [88]. Procedurally, this analysis will involve three steps: coding, within-case analysis, and cross-case analysis. In the first step, all qualitative data will be coded in NVivo. Our initial codebook will be informed by constructs identified in Fig. 1 and the results of phase 1 data collection but may subsequently be refined to include emergent constructs identified from the data.

In the next step, we will conduct within- and cross-case analyses. Specifically, coded data will be analyzed to identify themes in the coded data for each construct, the degree to which these themes emerge in the data (“strength”) and the degree to which each construct positively or negatively affects evidence use by staff (“valence”). We will also assess the degree to which observed relationships within and across cases are consistent with the conceptual model outlined in Fig. 1 [89].

Finally, phase 2 qualitative data will be calibrated and analyzed using QCA. Our objective in applying QCA will be to identify combinations of factors associated with high levels of staff research use and low levels of staff research use. Given the limited number of cases in our sample, limited diversity may prevent us from identifying meaningful combinations of factors associated with the desired outcomes. If that is the case, we will forego QCA and rely purely on the within- and cross-case analysis results. Results will be shared with participating agencies and used to refine the conceptual model in Fig. 1.

## Discussion

Prior research has identified numerous barriers to evidence use by practitioners. Research evidence can be costly for practitioners to access. The quality of available evidence and its perceived relevance to local organizational and practitioner needs can also vary considerably [40, 43, 90]. In particular, many EBTs are developed and tested with specific client populations in relatively resource rich settings; however, the contexts in which practitioners are expected to translate these EBTs are often significantly more heterogeneous in terms of available resources, client characteristics, and supportive infrastructure [91–93]. Strategies for beginning to address such concerns include the use of hybrid research designs that simultaneously assess program effects and implementation and the development of processes that allow for structured (rather than ad hoc) adaptation of EBTs to local contexts [94, 95]. However, equally critical for sustainment, scale-up, and spread of evidence to lower resource settings are a better understanding of the contexts in which evidence is being used and the extent to which effective strategies for facilitating evidence use may vary across these contexts.

This study identifies organizational supports (e.g., technical infrastructure, knowledge management infrastructure, linkage and exchange efforts) being used by private child and family serving agencies to promote evidence use. The study also examines the contexts in which these supports are more prevalent, identifies multilevel factors driving agency investment in these supports, and explores the conditions under which these supports may affect evidence use at different levels of the organization. Key strengths include the study’s focus on private child and family serving agencies, who play a critical role in delivery of publicly funded behavioral health and/or human services but are under-examined in the literature; the use of a multi-state sample that allows for systematic examination of how different institutional and market factors influence agencies’ support for evidence use; and the use of a configurational comparative approach to inform analyses, which will allow for identification of solutions that better reflect the reality of complex organizational phenomena.

In all states, public and private agencies are challenged to use evidence to improve organizational performance, including child and family outcomes [96–99]. Results will provide insight into strategies that may be effective for scaling evidence use at multiple levels of the organization and across different institutional environments and, ultimately, improving outcomes for vulnerable children and families.

## Abbreviations

Alliance: Alliance for Strong Families and Communities
EBTs: Evidence-based treatments
QCA: Qualitative comparative analysis
